# Sound of Daily Living Identification Based on Hierarchical Situation Audition

**DOI:** 10.3390/s23073726

**Published:** 2023-04-04

**Authors:** Jiaxuan Wu, Yunfei Feng, Carl K. Chang

**Affiliations:** 1 School of Information Science and Engineering, Shenyang Ligong University, Shenyang 110180, China; 2 Science and Technology Development Corporation, Shenyang Ligong University, Shenyang 110180, China; 3 Department of Computer Science, Iowa State University, Ames, IA 50011, USA

**Keywords:** computer audition, activity of daily living, situation-aware, smart healthcare, sounds of daily living

## Abstract

One of the key objectives in developing IoT applications is to automatically detect and identify human activities of daily living (ADLs). Mobile phone users are becoming more accepting of sharing data captured by various built-in sensors. Sounds detected by smartphones are processed in this work. We present a hierarchical identification system to recognize ADLs by detecting and identifying certain sounds taking place in a complex audio situation (*AS*). Three major categories of sound are discriminated in terms of signal duration. These are persistent background noise (*PBN*), non-impulsive long sounds (*NILS*), and impulsive sound (*IS*). We first analyze audio signals in a situation-aware manner and then map the sounds of daily living (SDLs) to ADLs. A new hierarchical audible event (*AE*) recognition approach is proposed that classifies atomic audible actions (*AA*s), then computes pre-classified portions of atomic *AA*s energy in one *AE* session, and finally marks the maximum-likelihood ADL label as the outcome. Our experiments demonstrate that the proposed hierarchical methodology is effective in recognizing SDLs and, thus, also in detecting ADLs with a remarkable performance for other known baseline systems.

## 1. Introduction

As we continue developing a large array of computing services as part of the Internet of Things (IoT), it is imperative to first acquire a deeper understanding of human activities of daily living (ADLs) [1] for those receiving such services. For example, sensor data enable an IoT system to monitor elderly rehabilitation [2]. Human ADLs are intimately embedded in the physical space, and our very nature makes us socially engaged. This paper focuses on the automatic identification of ADLs pertaining to IoT as an important basis to improve the quality of experience (QoE) and promptness when providing computer-based services in various forms.

ADLs are things people normally do during their day-to-day routines, including eating, bathing, dressing, grooming, working, homemaking, and leisure. The ability or inability to perform ADLs can be a very practical measure of a person’s capabilities when suffering from certain types of medical disorders [3]. Oftentimes, in a health care facility, with the help of observations by nurses and self-reporting by residents, professional staff collect and manually enter ADL data into a documentation system.

Some smart healthcare technologies have already been applied to detect ADLs. For instance, flexible touch sensors, embedded in a mattress, can detect the time when a bed is in use. Other examples include door contacts, infrared sensors, button sensors that detect the use of utilities, surveillance cameras that capture the ADLs after video processing, and microphones set into the ceiling that record in real time [4]. There are drawbacks to these conventional solutions [5].

Many devices are both expensive and labor intensive as they require installation in every room where sensing is required. Furthermore, these methods can infringe on privacy. Smartphones can act as an intermediary between body sensors and the web [6]. We developed an ADL Recorder App [5,7] to recognize ADLs with sensor fusion from multiple different sensor sources in one smartphone. It supports the early detection of abnormal situations, remote monitoring, and promotion of safety and well-being. This App and the back-end recognition system are among the first mobile applications to recognize ADLs using only a single standard smartphone.

We use the microphone on a smartphone as one kind of sensor to record surrounding sounds. Oftentimes ADLs create sounds, and sounds are reflections of the elements in the environment as well. The sound that an object makes can be mapped back to the action the human took. For example, a fan turns on when someone flips its switch, and the rotating fan blades transmit sound into the environment.

The smartphone is able to record the sounds that are audible to human ears. This study aims to recognize ADLs through the sounds of daily living (SDL), where ADLs are from the perspective of human and SDLs are from the perspective of physical objects. We assume that SDL represents a portion of the scene of ADL. In this case, our App interacts with human behaviors and transfers data over a network to the ADL Recognition System [7], which then sends the ADL history information back to reviewers, thus, completing the human-to-human and human-to-computer interaction in the IoT setting.

Some household event sounds can be weak, such as the sounds of tearing aluminum foil and flipping a newspaper. In order to pinpoint such ADL events, it is necessary to filter out blank periods and trivial sound segments in order to reduce the computational load. To achieve this, we present a novel hierarchical situation-based architecture, combining several audible event detection and identification techniques, specifically for household sound classification.

In order to improve the recognition accuracy, we first detect and extract audible events out of the situations from a recording session and train those audible events as acoustic models afterwards rather than training signals in one session as a whole. We consider one session of compound sound as an audible situation (see Section 4.3) in our ADL study. The benefit is the ability to recognize the pure audio clips at the same level, so that the key parts stand out in the audio clips. Subsequently, our recognition rate gains better performance. A novel fragmentation technique is introduced in our system. The fragmentation technique pinpoints when acoustic events are happening and extracts those clips for better recognition. This fragmentation technique can serve as a general processing step plugged into other algorithms, which is not dependent on the types of acoustic features and classifiers.

The remainder of this paper is organized as follows. In Section 2, we discuss some of the prominent previous works and underline their research scope and approaches. Section 3 illustrates three major sound categories in terms of the duration of audible parts. Section 4 describes the fundamental relationship between the sound waveform domain and situation-aware sound domain. After generally describing the system in Section 5, a new probability-based audible event recognition algorithm is elaborated in Section 6 and Section 7. We present the experiment setup, exploring the accuracy improvement by AA-based fragmentation in other baseline classification systems in Section 8. Finally, Section 9 summarizes the present study and concludes with future work.

## 2. Related Work

In regards to environmental sound classification, the objectives of existing research show diverse goals. Furthermore, a variety of combinations of acoustic features and classifiers have been experimentally used. We summarize the performance of related environmental sound-recognition techniques in Table 1. Differences of the audio tested in the literature include the aspects of duration of the testing audio excerpts, events and places (e.g., traffic, bathroom, cafe, and hallway), and sound classes. Moreover, the classification outcomes can be expressed in different levels.

For example, [8] investigated both event-level (e.g., car, bus) and occasion-level (e.g., public places and home) contexts. Furthermore, [9] classifies five environmental noise classes (babble, car, bus, factory, and street) using line spectral features and a Gaussian classifier, on a frame-by-frame basis using short segments (e.g., 20 ms) of the signal. The noise analyzed in [9] sometimes represents background sound (traffic), sometimes event sound (bus), and sometimes simultaneous conversations. All kinds of noise are fed into such classifiers as a whole, which yields the overall error rates ranging from 20% to 35%.

**Table 1:** Comparison of the recognition accuracy in previous works.

Ref.	Features	Classifier	Recognition Rate
[8]	MFCC	GMM; 1-NN; HMM	system 27 contexts = 58%; 12 MFCC + GMM = 63%; listener = 69%; system high-level = 82%; listeners high-level = 88%
[9]	Line spectral	QGC; DTC	86.4%; 88.1%
[10]	MP+MFCC	k-NN; GMM	system = 83%; listeners = 82%
[11]	LPCC	Discrete HMMs	system = 90%+; listeners = 91.8%
[12]	FFBE;ZCR; STE;4SBE;SF;	SVM	81%
ours	FFT	Segmentation; Fragmentation; GMM+HMM	basic level = 90%+; sub-category = 80%+

Note: MP: matching pursuit; QGC: quadratic Gaussian classifier; DTC: decision tree classifier; LPCC: linear predictive cepstral coefficients; FFBE: 16 frequency-filtered log filter-bank energies; ZCR: zero crossing rate; STE: short time energy; 4SBE: 4 sub-band energies; SF: spectral flux; and FFT: fast Fourier transform.

There are still some general issues that have been overlooked in previous works. Most of the previous studies (e.g., [12,13]) incorporated experiments using isolated acoustic events in silent environments. However, the contexts in the real world are more complex.

The audio clips for training in the reviewed literature are compound sounds mixing environmental sound and behavioral sound. For example, the background noise of a particular environment composed of many sound events was explained in [10], which did not take each individual constituent sound event into account but instead the many interweaving properties of each environment. Unfortunately, computer-based sound detection generally lacks the selective attention ability of human ears. The raw audio can capture the general properties of all sounds including background, music, speech, and those caused by audible actions. It, therefore, results in a large error rate for dealing with a mixture of overlapping audio events.

Instead of separately estimating a GMM for each audio segment, ref. [14] estimates a GMM for each audio segment by adapting the parameters of a universal background model (UBM). Thus, UBM becomes a trained GMM to represent all types of audio. However, the whole “recording session” (hereafter, shortened as “session”) contains many audio types incurring much interference, resulting in inaccuracy in model training.

We define audio classification resolution (ACR) to quantify the extent of details that the classification system resolves from the audio excerpts. Higher resolution means more audio detail. For instance, provided that making a pizza is one of the basic-level actions under the encompassing cooking class, then the set of {kitchen, apartment} is the super-category with a lower resolution, representing an environment where the actions are happening. A higher ACR of cooking’s sub-category can be obtained through recognizing more basic elements of more details, such as placing a pan onto an oven, switching on a heater, and running water. However, the datasets selected by a number of previous works [9,10] provide audio clips in different ACRs for the classification work, thereby, resulting in lower recognition accuracy.

## 3. Sound Categories

The terminologies for acoustic event classification are somewhat confusing, such as “environmental sound [10]”, “background noise [9]”, and “environmental noise [11]”. While, sometimes, a commonly used term may indeed represent the same issue, oftentimes it may not. For example, event sounds and environmental sounds can be misrepresented. In this paper, we need to first clearly define the sound categories in order to convey the essential concepts introduced in this paper.

Our living environment is full of various types of sounds every day. At home, we experience sound from movement, conversation, television and radio, the usage of household appliances, etc; on the road, we are exposed to noises, such as construction, traffic, and cars.

In this research, we broadly categorize the sounds commonly encountered in daily life into three types in terms of time duration and amplitude. They are persistent background noise (*PBN*), non-impulsive long sound (*NILS*), and impulsive sound (*IS*).

### 3.1. Persistent Background Noise

Persistent background noise (*PBN*) is a kind of perpetual background sound. Such continuous sound always lasts over an extended period of time, such as noise generated in a printing center and the sounds from a heater fan, air conditioner, mist vaporizer, vacuum cleaner, and kitchen hood fan.

**Definition** **1.** *In one time period of SDL audio signal S, the PBN = {$S_{i}$ $|\;D_{i}$ == the length of $S_{i}$ and average($A_{i}$) ≤ $upperbound_{A}$}, that is to say, the PBN is always long-lasting in the environment, where $S_{i}$ is a certain sub-audio signal in S, $D_{i}$ is the duration of signal $S_{i}$, $A_{i}$ is the amplitude of signal $S_{i}$, average($A_{i}$) is the average of $A_{i}$, $upperbound_{A}$ is the upper bound of amplitude of S, which can be calculated by Equation* (1).

(1) $$20\mathrm{lg}\frac{upperbound_{A}}{max(the\;amplitude\;of\;S)}dB\leq -12dB$$

### 3.2. Non-Impulsive Long Sound

The second type of sounds refers to the non-impulsive long sounds (*NILS*), such as an explosion, bell ringing, and these are not very long yet are audible. Such non-impulsive sounds are not always hidden behind the background *PBN* and are longer than an impulsive sound. In general, *NILS* comes from either human behavior context or the environmental context [15]. Mostly, *NILS* occurs along with some events, thus, we can determine audible actions or events involving humans, environments and their interactions. For example, running water sounds indicate that someone is washing, and sounds from moving utensils are clues of people’s actions. This is an example indicating that SDLs can be mapped back to ADLs.

**Definition** **2.** *In one time period of SDL audio signal S, the NILS = {$S_{i}$*∣*$Threshold_{D}$$\leq D_{i}\leq$ the length of $S_{i}$ and $average\left(A_{i}\right)\geq upperbound_{A}$}, where $Threshold_{D}$ is the threshold of the time duration, generally takes [2,12) seconds.*

In this research, we use these features to conjecture about people’s activities of daily living (ADL).

### 3.3. Impulsive Sound

Impulsive sound (*IS*) refers to a category of unexpected, almost instantaneous (thus, impulse-like) sharp sounds (such as clicks and pops). Such an intense “impulse” sound usually occurs once in a very short time interval, atomically lasting less than 1 s. Furthermore, some of these are punctuating, such as system noise (e.g., electromagnetic interference). The frequency energy of some impulsive sounds is over a large portion of the acoustic spectrum, such as a hammer blow or hand clap.

**Definition** **3.** *In one time period of SDL audio signal S, the IS = {$S_{i}|0\leq D_{i}\leq Threshold_{D}$ and $average\left(A_{i}\right)\geq upperbound_{A}$}, where, normally, $Threshold_{D}\leq 1$ second. Mostly, IS happens within a NILS session.*

When someone exerts a force on an object, we assume that the reaction of the object immediately emits an audible signal and lasts for a period of time. Furthermore, the lasting part gives the new state of the object. Thus, *NILS* often changes an object into a new state. For instance, water starts running after someone turns on the faucet, and the *NILS* of running water represents the state of *faucet turned on*. The *NILS* ends when the state of the faucet reverts to off.

**Figure 1:**
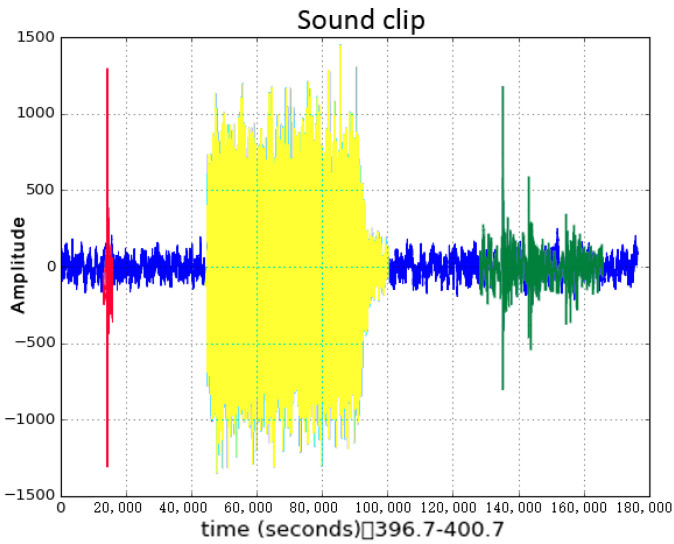
Waveform of a short audio excerpt.

In the real world, these three major sound categories naturally interweave with each other, because events happen together as time progresses. Figure 1 is a waveform of a sound excerpt. The whole audio file was recorded in a kitchen, which contained many featured sounds as the subject was cooking a meal. In this scenario, the person cooked as usual, conducting many actions, such as placing a baking pan onto an oven and taking a large bowl from a drawer.

We extracted a four-second sound clip out of the entire cooking audio file and computed the wave diagram. In Figure 1, the blue part is background noise, which belongs to PBN. The green part, from stirring eggs, is one kind of NILS. The yellow part of the sound file is bell ringing, lasting around one second, which also belongs to NILS. Finally, the red part of the sound is from hitting a spoon on a dish, lasting around ten milliseconds, which belongs to IS.

## 4. Hierarchical Situation-Aware Audition Analysis

A situation in this work is a sequence of actions under a certain environment. In the real world, human behaviors exist in their living environment. From the sound domain perspective, sounds of people’s actions occur in an environment where a diverse set of sounds are pervasive. When a user is taking their actions in sequence, the sound happening simultaneously reflects the changing situations in progress—that is, situation transition. As such, one action may introduce new auditory information into the earlier auditory environment.

Our work can reveal the sequence of actions by means of establishing situation-awareness of auditory information, by perception of the human behavioral contexts and environmental contexts. In this section, we leverage the sound happening along with situation transition to hierarchically decompose one session of *audible situation* into several *audible events* and *audible actions*, discovering the situation information based on auditory characteristics. [16] defined that a situation can be atomic, logical composite or temporal. Here, in relation to [16], an *audible action* is considered as an atomic unit, and an *audible event* is considered as logical composite or temporal.

### 4.1. Audible Action (AA)

**Definition** **4.** *An AA is an atomic action performed by a person and is mostly accompanied by a perceptible sound in short time intervals.*

Our project focuses on key nontrivial *AA*, such as an unpredictable door slamming, stepping heels, and snapping fingers. Usually, an *IS* corresponds to one *AA*, where one *AA* has only one crest of the wave. While, in reality, there can be a certain single *AA* that contains more than one crest of the wave, such as a jar lid that continues vibrating after being thrown on a table. In such cases, we can consider each crest as one micro-AA.

### 4.2. Audible Event (AE)

**Definition** **5.** *An AE is the representation of the sound effect due to human presence or due to the sound of objects and nature in the indoor environment.*

An *AE* may contain one *AA* by one person or a number of *AA* from more than one person. This work focuses on the single agent domain. We aim to identify both timestamps and types of events in an audio stream.

(2) $$AE=\left(\cup_{i}AE_{i}\right)\cup \left(\cup_{j}\bar{AE_{j}}\right)$$

where $\cup_{i}AE_{i}$ represents the set of AEs in the indoor environment, and $\cup_{j}\bar{AE_{j}}$ represents the set of indoor environment contexts.

An *AE* comprises a sequence of *AA*s along the time domain, related to the human behavioral context. A specific example AE is *frying eggs*. This entails multiple instances of a spatula touching a pan, each of which is an *AA*. This AE is composed of multiple ISs. Each touching *IS* is an AA and the session of *IS*s burst is an AE. The first type of *AE* is the continuous sequence of *AA*s, which reside in one session of *AE*.

(3) $$AE_{i}=sequence\left\{AA_{i}^{1},AA_{i}^{2},AA_{i}^{3},\ldots ,AA_{i}^{j}\right\}$$

Another type of *AE*, related to the environmental context, is an audible state of an object. For example, running water is an audible state of a faucet, and ringing is a state of a bell.

(4) $$\bar{AE_{j}}=audibleState\left(object\right)$$

Note that audibleState is a mapping from the state variable of an object to a set of state values, such as {busy, idle} for the object being a faucet, or {ringing, silent} for the object being a phone.

### 4.3. Audible Situation (AS)

In terms of ADL recognition, it often relates a human subject’s actions to the daily goals to be accomplished, such as personal hygiene, feeding, and entertainment. Moreover, goals can be decomposed into several sub-goals.

In general, much of the SDL information is composed of several simultaneous *AE*s that lead to a complex audio environment and increases the difficulty in audio retrieval and analysis. An *AS* is even more complex as it is normally mixed with all three different kinds of sounds. Thus, we give the following definition.

**Definition** **6.** *If there is at least an AE happening in a named situation (i.e., situation pertinent to a particular application), then we say this is an AS.*

(5) $$AS=\left\{A,E\right\}=\cup_{i}AS_{i}=\cup_{i}{\left\{\cup_{j}AE_{i}^{j},E_{i}\right\}}_{\Delta t}$$

*where each AE completes a sub-goal for the time being. $\cup_{i}AS_{i}$ represents a set of AS, $\cup_{j}AE_{i}^{j}$ represents a set of AE in $AS_{i}$, A is a set of the users’ actions to achieve a goal, and $E_{i}$ is a set of context values with respect to a subset of the environmental context variables during a period of time Δt when the sequence of As is completed.*

Now, that goals can be decomposed into several levels of sub-goals, AS can be also decomposed into several levels of $AE_{i}$ and $E_{i}$ as appropriate. Suppose a named $AS_{i}$ of *making salad in the kitchen* contains many higher-ACR AE sessions, such as taking tomatoes from a plastic bag, cutting carrots, and stirring salad. Furthermore, the environmental context E is in the kitchen. Therefore, we can predict the auditory scenes, in reference to a location with different acoustic characteristics, such as a printing center, on a bus or in a quiet hallway, from an *AS*. On the scale of ACR, *AA* has a higher resolution than *AE*, which similarly has a higher resolution than *AS*.

Our belief is to utilize sound information as much as possible. Environmental context helps to predict the place and environment that subjects reside in. In any *AS*, environmental contexts can be retrieved by background sound or $\bar{AE}$, and behavioral contexts can be captured and recorded as the sound snippets of *AE*.

## 5. System Overview

In [10], classification was conducted across various audio environments, such as restaurant, street, thundering, and sea waves. From our perspective, the environments are not in the same ACR. ACRs in different levels correspond to different sound categories as previously defined. *Thundering* belongs to *NILS*; *sea waves* belong to *PBN*; *restaurant* is an *AS* which composes of the noisy, quiet, or music-laden *PBN*s. It also includes some *NILS* in the *AE* level, such as *moving chairs* and *slamming a door*. It even includes many sounds of *spoon/cup jingling*, which is an *IS* in the *AA* level.

As we have already clearly distinguished between environmental sounds and behavior sounds, the major task of this project is to extract *NILS* and *IS* out of a long audio sample and recognize the actions under a certain situation.

In this work, we first accomplished the task of AEs detection and extraction from a long SDL audio file with Algorithm 1, which is an AS. The acoustic feature used is the first coefficient in a Mel-scale frequency cepstral coefficient (MFCC) vector, as it reflects the energy information. Subsequently, in order to improve the recognition accuracy of AEs, a technique of fragmenting AE files into several AA snippets was applied using Algorithm 2.

**Figure 2:**
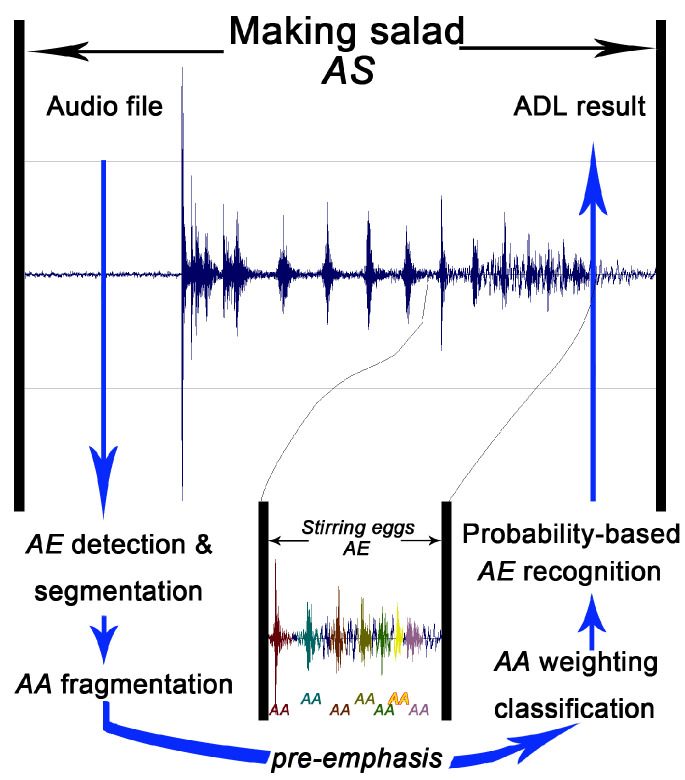
System diagram of hierarchical situation audition.

Pre-emphasizing the high-frequency region of AA samples was engaged to enhance the acoustic characteristics. The combination of Gaussian Mixture Model (GMM) and Hidden Markov Model (HMM) classification computes using each AA snippet and yields a potential type outcome. At last, the synthesized AE classification is determined by the AA type with the largest probability. The schematic diagram of the proposed methodology is shown in Figure 2.
**Algorithm 1:** AE detection and extraction algorithm
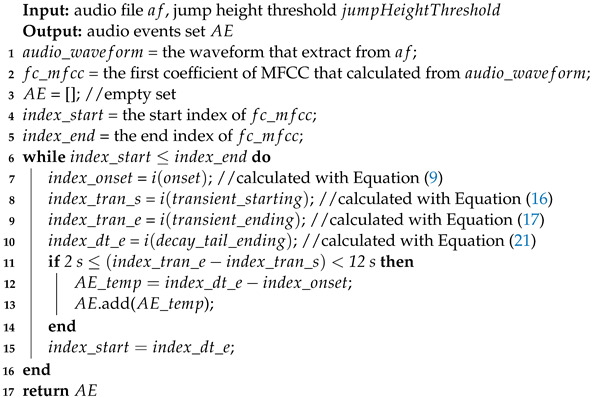

**Algorithm 2:** AE detection and extraction algorithm
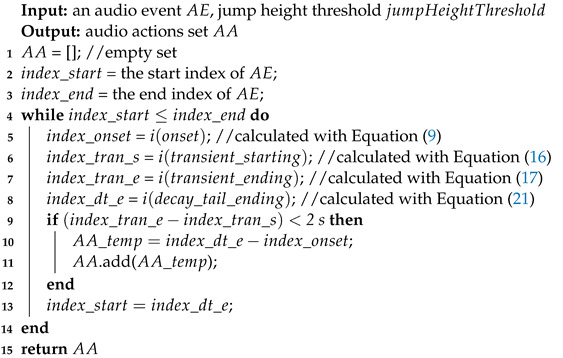


## 6. Audible Actions/Events Detection and Extraction

Mel-scale frequency cepstral coefficient (MFCC), as a kind of acoustic features, is commonly used for speech/speaker recognition because it considers human perception sensitivity with respect to frequencies. Typically, studies gain better performance by leaving out the first coefficient of MFCC (fc_mfcc). This is because the first coefficient relates to the magnitude of power [17], which is usually much larger than the other coefficient, thus, causing distortion in computation. Our research happens to benefit from using fc_mfcc to extract and segment valuable parts from a long audio file. Therefore, MFCC features were used for all steps of event detection, audio segmentation, and feature extraction together.

The extraction process was operated on the first coefficients of the MFCC (fc_mfcc) array. The key points of fc_mfcc were converted into corresponding positions in a real wave file, according to which AA / AE clips were extracted. Every element in this array corresponds to a frame from the audio clip.

### 6.1. Stages of an Atomic Audible Action/Event

**Figure 3:**
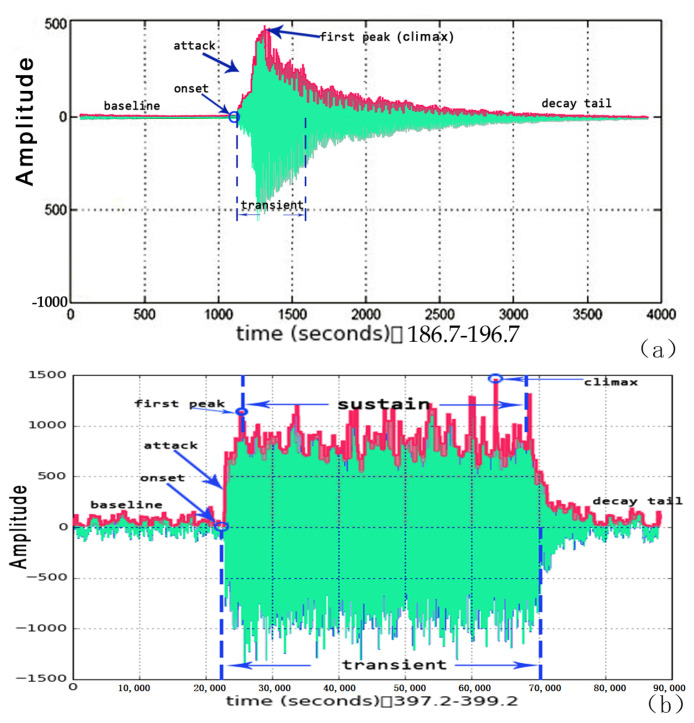
(**a**) “Baseline”, “onset”, “attack”, “first peak”, “climax”, “transient”, and “decay tail” in an audible action AA. (**b**) Stages in a bell ringing sound ($\bar{AE}$), containing a “sustain” stage.

An isolated *AA* is composed of six stages: baseline, onset, attack, peak, transient and decay tail, as the waveform of a simple case shown in Figure 3a.

1. The baseline is the base sound prior to and posterior to the *AA* session, whereas v(baseline) is the value of base sound, whose acoustic features are used to establish environmental context.
2. The onset of the *AA* is the earliest moment when the *AA* occurs, which coincides with the starting point of the transient.
  The height of a frame is its amplitude difference beyond the baseline.
  (6) $$h\left(f_{i}\right)=v\left(f_{i}\right)-v\left(baseline\right)$$
  where *i* is the index of a frame, $h(f_{i})$ denotes the referenced amplitude level of the *i*-th frame, and $v(f_{i})$ denotes the amplitude of *i*-th frame.
  The jump height of a frame is the rising amplitude difference compared to the previous frame.
  (7) $$\begin{array}{c} \Delta h\left(f_{i}\right)=h\left(f_{i}\right)-h\left(f_{i-1}\right),i=1,2,\ldots ,framesize. \end{array}$$
  (8) $$\begin{array}{cc} & F\left(i\right)=\Delta h\left(f_{i}\right)-jumpHeightThreshold,\\ & i=1,2,\ldots ,framesize. \end{array}$$
  where jumpHeightThreshold is a threshold for onset detection. The larger jumpHeightThreshold is, the fewer number of points of interest are obtained. It iterates through each frame and computes F(i), and the index of the first frame makes F(i)≥0, where the onset is detected. Thus,
  (9) $$\begin{array}{cc} & \left(\Delta h\left(f_{onset-1}\right)<jumpHeightThreshold\right)\\ & \mathrm{and}\;(\Delta h\left(f_{onset}\right)\geq jumpHeightThreshold) \end{array}$$
3. The climax is the maximum amplitude within the whole *AA* session.
  (10) $$i\left(climax\right)={\arg\max}_{f\in S}\left(fc\_mfcc\left(f\right)\right)$$
  where i(climax) is the index of the climax in the *first coefficient in MFCC* vector within the whole *AA / AE*; and *S* is the set of MFCC frames over the whole *AE* session.
  (11) h(climax)=v(climax)−v(baseline)
  A large climax reflects when a significant pitch occurs, so that human ears can easily detect and recognize those. On the other hand, from the audition computation perspective, those larger amplitude regions with larger power have more distinct characteristics.
4. The peak is the highest point over a certain region.
  (12) $$i\left(peak\right)={\arg\max}_{f\in \Delta S}\left(fc\_mfcc\left(f\right)\right)$$
  where ΔS is the fc_mfcc of a small limited excerpt in the whole AE session, ΔS⊆S.
5. For an *AA* or *AE* signal, the attack of the *AA* is the process during which the amplitude envelope increases from the baseline to the first peak.
  (13) i(first_peak)=min{i(climax)}
  (14) $$\left\{\begin{array}{ccc} i(attack\_starting) & = & i(onset)\\ i(attack\_ending) & = & i(first\_peak) \end{array}\right.$$
6. The period of transient can be reliably detected after onset. The process of attack is embedded within the transient, whose interval begins at onset, ends until the amplitude decreases to an attenuating ratio.
  (15) $$20\log\frac{h(transient\_ending)}{h(climax)}=G_{dB}$$
  where $G_{dB}$ is the amplitude ratio or gain in dB, usually being set as −6 dB.
  (16) i(transient_starting)=i(onset)
  From Equation (15), the index of transient ending is
  (17) $$i\left(transient\_ending\right)=\arg i(h\left(peak\right)\cdot 10^{G_{dB}/20})$$
  The transient ending comes after the peak.
  (18) i(transient_ending)>i(peak)
  The signal in the transient stage plays the most significant role in retrieving representative acoustic features.
7. An isolated $\bar{AE}$ may contain sustain within the transient interval. The sustain exists when within a time duration $T_{sus}$, the amplitude levels are always maintained upon a certain level, displayed in Figure 3b. The $T_{sus}/t$ gives the number of MFCC frames, where *t* is the frame-blocking size.
  (19) $$\sum_{j}^{j+\frac{T_{sus}}{t}-1}h\left(j\right)\geq \frac{T_{sus}}{t}*jumpHeightThreshold$$
8. The decay tail is a slow decaying period after the transient of the sound session. Furthermore, the lengths of decay tails vary. Some signals even have very short decay tail, almost reducing sharply.
  (20) $$\begin{array}{cc} & L(offset)=\\ & [v\left(climax\right)-\sum_{f=i_{t}}^{i_{t}+T_{sli}}\frac{v(f)}{T_{sli}}]-\left(1-\epsilon \right)*h\left(peak\right) \end{array}$$
  where $i_{t}$ is i(transient_ending)+1+offset, and offset is used for deriving the index when the L value begins to be positive.
  (21) $$i\left(decay\_tail\_ending\right)=i_{t}+T_{sli}$$
  where $T_{sli}$ is the sliding window size, and we compute the average amplitude in this window. Note that ϵ in Equation (20), ranging from (0,$-G_{dB}$), adjusts the dropping height from the peak to the decay_tail.

### 6.2. *AE/AA* Extraction Algorithm

At first, a fc_mfcc vector is computed from an audio waveform of a long audio file. To locate the onset and end of decay of one *audible action / event*, the jumpHeightThreshold is set to adjust the detection sensitivity for Equation (8). Equation (21) is applied to determine the ending of an *AA* or *AE*.

Another fast *AE* extraction method is locating onset and setting a fixed length of *AE* session as a parameter stepLength, which is used to confine the parts from onset to decaying tail within one session. Note that fewer AE segments are extracted with a larger stepLength.

Currently, there are a number of audio recognition systems using Hidden Markov Models (HMMs) to deal with the temporal variability, because HMM has advantages to render acoustic characteristics. A Gaussian Mixture Model (GMM) takes audio samples as scattered dots, measured from an acoustic perspective, including much non-speech information. However, GMM totally lacks sequence information that can be used to produce semantic information on acoustic feature vector. It is, therefore, applicable for non-speech sound recognition problems.

A simple Gaussian model is unlikely to be adequate since the feature (MFCC) data are unlikely to follow a simple normal distribution; a mixture of Gaussians is more feasible, and this is commonly used within HMM. Consequently, we may fit each state of HMM into a short window of frames with coefficients representing the acoustic input and train data into Gaussian mixture models. Thus, in this work, GMM is used for reflecting static patterns, and HMM is used for training sequential patterns in the audio clips.

In this research, sound recognition is to single out various *AE* types, such as the sounds of running water and chopping vegetables. In the *AE* domain, each type of *AE* sound varies in terms of rhythm, beats, repetition, object texture, etc. We assume that a combination of techniques (GMM and HMM) screening each *AE* sound at different granularity levels can help to identify *NILS* and *IS*. The details of the AE / AA extraction algorithm are shown in Algorithms 1 and 2.

## 7. Probability-Based *AE* Recognition Algorithm by *AA* Fragmentation

Instead of examining a large area of surface texture, a human is able to distinguish glass from wood by a glimpse over a small area, even to the solution level, such as surface texture. This is also true in the audio domain. Thus, similarly, if the audio acoustics are relatively uniform, they can be classified by a short audio excerpt. Thereafter, we introduce a fragmentation technique.

**Fragmentation**: A session of *AE* is fragmented into several *AA*s based on waveform energy. The fragmentation allows computers to process the *audible event* with finer granularity. Likewise, people always pay more attention to sound that is difficult to discern. In the SDL domain, an atomic *AA* generally takes 40∼400 ms, which is difficult for the human auditory system to discern, while a computer auditory system is capable of it.

(22) $$length\left({\mathrm{AE}}_{i}\right)\geq \sum_{k=0}^{N-1}length\left({\mathrm{AA}}_{i}\left[k\right]\right)$$

where $AE_{i}$ denotes the *i*-th *AE* session; $AA_{i}\left[k\right]$ is the *k*-th *AA* fragment from the $AE_{i}$; and *N* is the number of *AA* fragments from the *AE*. Except for *AA*s, each *AE* session contains non-contributing parts of the baseline because they are from the environmental context.

**Figure 4:**
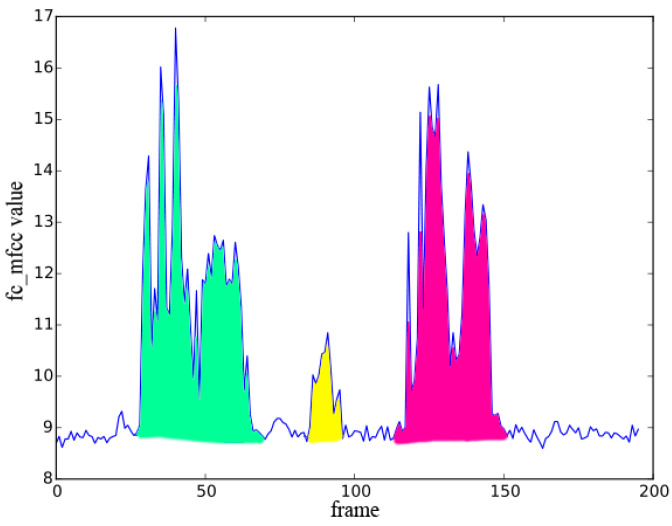
Three *AA*s of a knife hitting on a chopping board in a “cutting” *AE*.

***AA*** **weighting rule**: Long period *AA* clips with high amplitude have larger weights, because longer durations have richer acoustic features, and high amplitude gives it a larger range of amplitude variation and more maximal values. Thus, *AA* in the yellow region of Figure 4 has a smaller weight compared with the other two.

(23) $$energy\left(AA_{i}\left[k\right]\right)=max\left(fc\_mfcc\left(AA_{i}\left[k\right]\right)\right)$$

The energy function of an *AA* session is the maximum value (peak) of its first MFCC (fc_mfcc) vector. Here, we use maximum value to simplify the computation of energy.

(24) $$\begin{array}{c} i\left(peak\right)={\arg\max}_{f\in S}\left(fc\_mfcc\left(AA_{i}\left[k\right]\left[f\right]\right)\right),\\ f=0,1,\ldots ,framesize-1. \end{array}$$

fc_mfcc() has a length of framesize, which is the total number of frames of the *AA* session. *S* is the set of the atomic *AA* MFCC frames.

(25) $$\Delta \Delta (fc\_mfcc\left(AA_{i}\left[k\right]\left[i\left(peak\right)\right]\right))=0$$

The peak index is where the second derivative of fc_mfcc() equals zero.

(26) $$\omega_{k}=\frac{energy(AA_{i,j}\left[k\right])}{\sum_{k=0}^{N-1}energy\left(AA_{i,j}\left[k\right]\right)}$$

where $AA_{i,j}\left[k\right]$ denotes the *k*-th *AA* predicted as type *j* in the *AE* of session *i*, and the weight value ω corresponds to the amplitude level obtained.

**Probability-based*** **AE*** **prediction**: We compute the probability of potential *AA*s in one *AE* based on each *AA*’s accumulative portion in the *AE*.

(27) $$p_{i,j}\left[k\right]=\frac{length({\mathrm{AA}}_{i,j}\left[k\right])}{length({\mathrm{AE}}_{i})},\mathrm{and}\sum_{k=0}^{N-1}p_{i,j}\left[k\right]=1$$

where $p_{i,j}\left[k\right]$ denotes the probability of the *k*-th *AA* predicted as type *j* in the *AE* of session *i*;

(28) $$P_{i,j}=\sum_{k=0}^{N-1}\omega_{k}*p_{i,j}\left[k\right]$$

where *N* is the total number of *AA*s fragmented from the *AE* session; and $P_{i,j}$ is the probability of *i*-th AE session predicted as SDL type of *j*.

(29) $$\begin{array}{c} label\left({\mathrm{AE}}_{i}\right)=label\left(max\left(P_{i,j}\right)\right),j=0,1,\ldots ,M-1 \end{array}$$

Finally, we label the *AE* session with the *AA* tag of the maximal probability. The details of this algorithm are shown in Algorithm 3.
**Algorithm 3:** Probability-based AE recognition algorithm by AA fragmentation
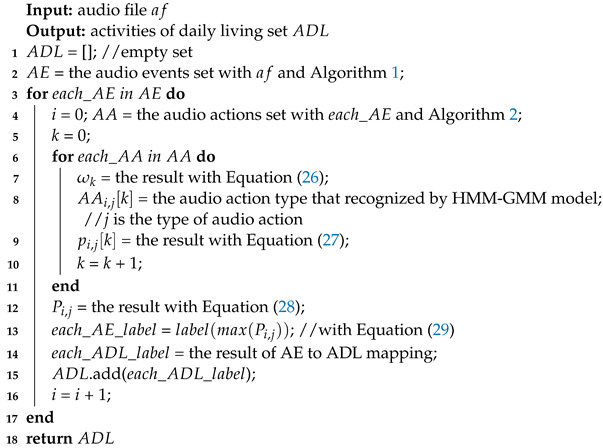


## 8. Experiment and Discussion

This study first applied the proposed hierarchical situation audition approach using featured household sounds, such as chopping, turning on the faucet, and placing a pan on a counter, where our algorithm gained high accuracy. The recordings of the sound clips that we used were from two datasets. One was recorded from multiple personal mobile phones with the ADL Recorder App [7], and the other was obtained from the Carnegie Mellon University Multimodal Activity (CMU-MMAC) Database [18], which recorded subjects performing the tasks involved in cooking and food preparation. In order to illustrate the classification performance in a more general manner, here, we present a full-scale experiment based on two recent standard open datasets, ESC [19] and TUT [20], which are commonly accepted by this research community.

### 8.1. Comparison with a Baseline Classification System

In this experiment, a publicly available dataset, ESC-10 [19], was selected for evaluation of the AA-based recognition algorithm. ESC-10 is a less complex standardized subset of 10 classes (dog bark, rain, sea waves, baby cry, clock tick, person sneeze, helicopter, chainsaw, rooster, and fire crackling), which has 40 clips per class.

Feature vectors were fed as input to three types of classifiers: k-Nearest Neighbors (k-NN), Random Forest Ensemble, and Support Vector Machine (SVM) with a linear kernel. As shown in Table 2, the results in the second to fourth columns are derived from testing the baseline machine classification provided by [19] on an Ubuntu 64-bit platform. The fifth column shows the results from running the GMM-HMM recognition algorithm without AA-based fragmentation, and the results on the sixth column were derived from running our AA-based recognition algorithm from MATLAB on an Ubuntu 64-bit platform. An iterative Expectation–Maximization (EM) algorithm to obtain a Maximum Likelihood (ML) estimate was applied to train the GMM model.

The ESC-10 dataset had an average classification accuracy ranging from 66.7% for the k-NN classifier to 72.7% for the random forest ensemble with SVM in the the sixth column (67.5%) and HMM in the eighth column (51.3%) [19]. When the AA-based recognition algorithm proposed in this paper was introduced, the recognition accuracy of the three methods improved to 76.4% (k-NN with AA-based), 80.2% (Random Forest with AA-based), 70.75% (HMM with AA-based) and 77.5% (SVM with AA-based), respectively.

In terms of acoustic features, the baseline system utilizes the first 12 MFCC coefficients, and zero-crossing rates were summarized for each clip with their mean and standard deviation across frames. Therefore, from the acoustic feature extraction step to the classification step, the system needs to deal with 15 features. However, the AA-based recognition system only extracts five dominant frequencies from standard FFT as the acoustic features, which largely reduces memory consumption.

In addition, the AA-based algorithm fragments audio clips in the preprocessing step, and the total size of the audio clips was largely reduced from 168 to 85.2 MBytes. Furthermore, the total sound length before AA fragmentation was 2003 s, and the total sound length after AA fragmentation decreased to 1011 s. Similarly, it saves nearly half of the memory in the feature extraction step.

### 8.2. AA-Based Fragmentation Contributes to Interior Sounds Classification

This experiment continues to use the baseline classification system, while the dataset contained only 10 classes interior domestic sounds selected from ESC-50 [19]. The types include clock alarm, can opening, door knocking, clock ticking, glass breaking, door wood creaking, mouse clicking, keyboard typing, washing machine running, and vacuum cleaner operating, with 40 clips per class.

The aim of this experiment was to investigate how the AA-based fragmentation technique contributes to the accuracy in the classification system when selecting the same acoustic features. Learning was performed on the datasets with a five-fold cross-validation regime.

In Table 3, the results in rows 1, 3, and 5 are the accuracy retrieved from the baseline classification system by using k-Nearest Neighbors, Random Forest Ensemble, and Support Vector Machine classifiers. The results in rows 2, 4, and 6 are the accuracy when applying AA-based fragmentation before learning and classifying. We can see that AA-based fragmentation slightly increases the accuracy for both k-NN and random forest classifiers, and this accounts for an improvement of 9.9% for the SVM classifier.

### 8.3. AA-Based Fragmentation Improves on Another Baseline System

In this experiment, we investigated how well AA-based fragmentation technique contributes to the acoustic scene classification accuracy for the baseline system provided in [20]. The audio dataset contained 15 types, including beach, bus, cafe/restaurant, car, city center, forest path, grocery store, home, library, metro station, office, park, residential area, train, and tram. Each type had 78 audio segments. Thus, it had 1170 audio clips in total.

The baseline system utilized a classical MFCC and GMM-based classifier. The first 20 coefficients of MFCCs were calculated for all of the audio, including the first coefficient. A Hamming window with 50% overlap was covered on 40 ms frames and 40 mel bands totally. Delta and acceleration coefficients were also calculated using a window length of nine frames, resulting in a frame-based feature vector of dimension 60. A GMM class model with 32 components was trained using an expectation maximization algorithm for each acoustic scene [20]. The testing stage used maximum likelihood decision among all acoustic scene class models. The classification results using the cross-validation setup are presented on the first row of Table 4. The overall performance was 72.5%, which was measured as the number of correctly classified segments among the total number of test segments.

Here, we have two-round experiments. In the first round, it generated 55,634 AA fragments out of the 1170 audio clips. In the second round, it generated 55,353 AA fragments in total. The classification accuracies in the AA-level and AE-level are shown from second to fifth rows in Table 4. By comparison, the AA-based technique improved the baseline accuracy from 72.5% to 77.0% for the first round and 77.6% for the second round. In addition, the accuracies achieved from folds have less deviation than those from the baseline system without AA-based fragmentation. Thus, the AA-based technique gained a high consistency of performance because it extracted the most valuable fragments out of the whole audio files.

## 9. Conclusions

Sound is pervasive in human environments, and ADL detection and identification are increasingly being accepted in the IoT era. This work reports our study on situation-aware audio analytics where typical smartphones were used to record sound, and the recorded sound was processed in a cloud-based backend recognition server. As the audio files were recorded and accumulated gradually, the multimedia datasets became increasingly large. Most parts of the audio files were trivial and recognized only once, thus, we proposed to shrink the datasets without affecting the recognition accuracy. We related the signals in sound waveform to the elements in situation-aware environments. As explained, our aim was to identify the SDLs by recognizing the audible event (AE) in a certain audible situation (AS).

Some researchers have worked on identifying sounds in smart homes; however, those sounds belong to single isolated *NILS*, most of which last 1∼5 s, such as a phone ringing and running water, which are considered as $\bar{AE}$. In this study, not only the single $\bar{AE}$ but also AE with multiple micro-AAs were analyzed and recognized. We presented a probability-based AA recognition classification method in Section 7 that is suitable for predicting *AE* by recognizing short IS fragments. We tested and validated that AA-based fragmentation was generally able to fit into other classification systems. This helped to improve the classification accuracy; moreover, it greatly saved on the ion memory consumption for both feature extraction and classification.

In the future, we will perform deeper analysis on the acoustic characteristics of some SDL types by exploring a new and stable algorithm R to recognize *PBN* and infer E. In addition, new technologies will be applied to compress and store the acoustic templates and models.

The significance of the presented work lies with the premise that humans living in the contemporary IoT social environment engage in various daily activities and oftentimes involve multiple agents. The automatic detection and identification of human activities can support researchers and developers to envision and develop novel services to improve the quality of experience and promptness for those who receive and consume such services. We are hopeful that this may help to stimulate interest for those like-minded researchers to produce more fruitful results in this critical emerging topic in IoT computing.

## Figures and Tables

**Table 2:** Recognition accuracy comparison of teh HMM-based AE recognition system and classification systems provided in [19].

ESC-10	k-NN	k-NN with AA Based	Random Forest	Random Forest with AA Based	SVM	SVM with AA Based	HMM	HMM with AA Based
Babycry	85.0%	87.5%	85.0%	86.7%	80.0%	86.5%	60%	80.0%
Chainsaw	40.0%	67.5%	52.5%	71.5%	40.0%	61.0%	45%	50%
Clocktick	27.5%	52.5%	47.5%	61.0%	50.0%	69.5%	45%	62.5%
Dogbark	67.5%	72.5%	77.5%	89.5%	82.5%	89.5%	5%	70%
Firecrackling	67.5%	74.0%	87.5%	92.7%	70.0%	77.5%	25%	85%
Helicopter	62.5%	79.5%	70.0%	74.5%	62.5%	68.3%	65%	67.5%
Personsneeze	92.5%	94.5%	82.5%	88.0%	75.0%	84.5%	82.5%	87.5%
Rain	60.0%	67.5%	65.0%	71.4%	62.5%	70.1%	62.5%	67.5%
Rooster	75.0%	77.0%	82.5%	87.5%	80.0%	88.5%	62.5%	85%
Seawaves	90.0%	91.5%	77.5%	79.5%	72.5%	79.7%	60%	72.5%
Average	66.7%	76.4%	72.7%	80.2%	67.5%	77.5%	51.3%	70.75%

**Table 3:** AA-based fragmentation contributes to the recognition accuracy in the baseline machine-classification system provided in [19].

Classi-Fier	Type	Fold1	Fold2	Fold3	Fold4	Fold5	Overall Accuracy
kNN	Baseline	60.0%	66.2%	61.2%	52.5%	57.5%	59.5%
	AA	52.8%	50.0%	59.1%	70.0%	70.0%	60.1%
RF	Baseline	60.0%	66.2%	67.5%	68.7%	60.0%	64.5%
	AA	58.3%	60.9%	55.5%	70.0%	80.0%	64.9%
SVM	Baseline	65.0%	61.2%	65.0%	56.2%	55.0%	60.5%
	AA	58.3%	60.0%	53.6%	80.0%	100.0%	70.4%

Note: RF: random forest.

**Table 4:** AA-based fragmentation contributes to the recognition accuracy in the baseline machine-classification system provided in [20].

	Type	Fold1	Fold2	Fold3	Fold4	Overall Accuracy
Based line		66.5%	68.9%	72.3%	82.2%	72.5%
round 1	AA	77.0%	77.3%	76.4%	76.9%	76.9%
round 1	AE	76.7%	77.0%	77.0%	77.2%	77.0%
round 2	AA	75.8%	76.3%	76.3%	76.3%	76.2%
round 2	AE	78.5%	78.0%	76.8%	77.1%	77.6%

## Data Availability

Not applicable.
